# Understanding Spatiotemporal Patterns of Biking Behavior by Analyzing Massive Bike Sharing Data in Chicago

**DOI:** 10.1371/journal.pone.0137922

**Published:** 2015-10-07

**Authors:** Xiaolu Zhou

**Affiliations:** Department of Geology and Geography, Georgia Southern University, P.O. Box 8149, Statesboro, GA 30460, United States of America; Peking UIniversity, CHINA

## Abstract

The growing number of bike sharing systems (BSS) in many cities largely facilitates biking for transportation and recreation. Most recent bike sharing systems produce time and location specific data, which enables the study of travel behavior and mobility of each individual. However, despite a rapid growth of interest, studies on massive bike sharing data and the underneath travel pattern are still limited. Few studies have explored and visualized spatiotemporal patterns of bike sharing behavior using flow clustering, nor examined the station functional profiles based on over-demand patterns. This study investigated the spatiotemporal biking pattern in Chicago by analyzing massive BSS data from July to December in 2013 and 2014. The BSS in Chicago gained more popularity. About 15.9% more people subscribed to this service. Specifically, we constructed bike flow similarity graph and used fastgreedy algorithm to detect spatial communities of biking flows. By using the proposed methods, we discovered unique travel patterns on weekdays and weekends as well as different travel trends for customers and subscribers from the noisy massive amount data. In addition, we also examined the temporal demands for bikes and docks using hierarchical clustering method. Results demonstrated the modeled over-demand patterns in Chicago. This study contributes to offer better knowledge of biking flow patterns, which was difficult to obtain using traditional methods. Given the trend of increasing popularity of the BSS and data openness in different cities, methods used in this study can extend to examine the biking patterns and BSS functionality in different cities.

## Introduction

Biking is gaining popularity in many cities, which brings health and environmental benefits [1, 2]. Biking for commuting and recreation is one promising approach to counter the trend of declining physical activity [3]. In addition, promoting bikes as means of transportation is associated with reduction in pollution and traffic congestion [4]. In the United States, based on short trip analyses from national household travel survey (NHTS), from 2001 to 2009, about half of all trips were within 4,828 meters (three miles). However, more than 70 percent of these trips involved automobiles [5]. Subtle change in short distance travel from driving to biking may therefore dramatically contribute to environment and public health [6].

The growing number of bike-sharing systems (BSS) in many cities does much to facilitate biking for transportation and recreation purposes [7, 8]. The increasing popularity of the bike-sharing systems has developed alongside technological changes. Most recent bike-sharing systems produce time and location specific data, which enables the study of travel behavior and mobility at the individual level [9]. The data also provide useful information for transportation planning and management [10]. However, because of their relatively short history of smart BSS, despite a rapid growth of interest, studies on biking behavior using massive BSS data, and the underneath mobility pattern, remain limited [11, 12].

**Fig 1:**
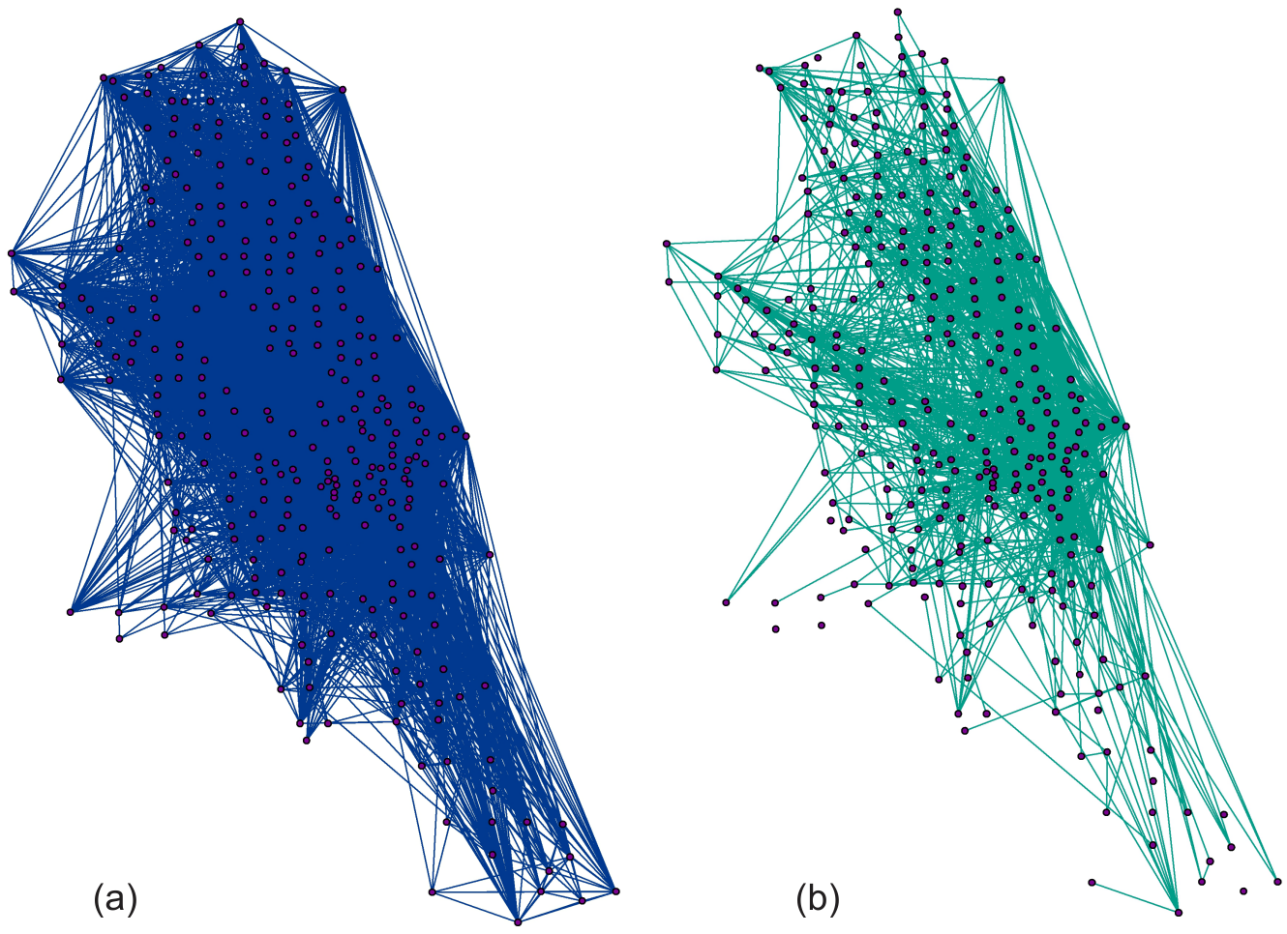
Ten percent and one percent subset of weekend trip map in 2013.

Previous studies that consider this topic leave several issues poorly addressed. First, understanding and visualizing patterns out of massive bike-sharing data is challenging. The cluttered display and overlaps of thousands of trips make it very difficult to extract informative patterns [13]. For instance, Fig 1 represents ten percent and one percent of bike trips on weekends in Chicago; the cluttered display of trips eclipsed the underneath patterns. Better trip analysis methods are required in order to discover and differentiate biking patterns during different time periods, such as weekdays, weekends, peak hours or even years. Second, since users can check-out bikes from one station, and return to any stations, bikes can be disproportionally distributed among different docks in the network. This may present a challenge; namely, that people may not be able to find a bike at certain stations because of empty docks, or they may experience difficulty returning a bike because of full docks. Few studies have investigated the spatiotemporal patterns of the over-demand for bikes and docks at different locations and times and the associated clusters. Third, most BSS in the US were developed in recent years. Furthermore, these systems’ trip data of was opened to the public even more recently. The Divvy bike-sharing systems in Chicago, for example, were launched in June 2013. Few studies have analyzed the system use and compared spatial patterns in 2013 and 2014.

Given this context, this study aims to answer two questions by analyzing rich amount of data generated by the bike-sharing system in Chicago: 1. How do bike flow patterns vary as a result of time, weekday or weekend, and user groups? 2. Given the flow patterns, what was the spatiotemporal distribution of the over-demand for bikes and docks in 2013 and 2014? This study contributes a better understanding of public biking mobility patterns. By using the proposed flow clustering based on community detection method, this study clearly demonstrates biking patterns by identifying major and representative clusters at certain windows of time, and filtering out noisy and unrepresentative trips. In addition, modeling over-demand patterns using hierarchical clustering helps to better understand the characteristics of bike station usage across all stations. The cluster profile analysis indicates the functional characteristics of each cluster. Ultimately, results can be used to maintain a balanced bike-sharing system, as well as design new biking facilities to promote active transportation.

### The Public Bike-Sharing System

Public Bike-Sharing has a half-century long history [14]; however, the popularity of the BSS remained low until recent decades. The capability of providing flexible mobility, as well as reducing emission and transportation costs increased the popularity of the modern BSS. By the end of 2013, there were about 0.6 million public bikes located in 600 cities across the world [15].

Many BSS studies were conducted in European cities. As of 2009, there were about 19 European countries operating bike-sharing programs [14]. Among these studies, analyzing the impact of the BSS on mobility was one research focus. For instance, French cities offered many early bike-sharing systems, including the Vélo’v in Lyon, in 2005, and the Vélib’ in Paris, in 2007. The percentage of trips by bikes increased from 1.0% in 2001, to 2.5% in 2007, in Paris, and from 0.5% in 1995 to 2% in 2006 in Lyon [16, 17, 18]. Another group of studies focused on the bike-sharing spatial usage patterns in cities, such as speed and pathways. In Lyon, France, previous studies analyzed BSS usage patterns and typology of cyclists using Vélo’v data [10, 19, 20]. In Barcelona, Spain, studies examined the station characteristics, such as spatial distribution and service patterns, of the Bicing program [21,22]. Some scholars have also considered the spatial inequality of bike-sharing programs and gender difference. Goodman and Cheshire investigated the BSS use patterns of women and residents of deprived areas, using usage data [1]. Beecham and Wood found that BSS usage patterns of female customers were very different from those of male customers using London’s Cycle Hire Scheme [9].

Asian cities also witnessed a fast development in bike-sharing systems, with ‘Nubija’ in South Korea, ‘C-Bike,’ and ‘YouBike’ in Taiwan being representative systems [23]. Zhang et al. analyzed the bike-sharing systems in terms of service, business model, and management in five cities in China [24]. Shaheen et al. conducted intercept surveys in Hangzhou to understand factors influencing bike-sharing adoption, and barriers to adoption [25]. Zhao et al. analyzed bike sharing travel patterns by gender and day of the week, and found significant variation in both variables [26].

Comparing the BSS in Europe and Asia, the BSS in the US was more limited prior to 2010. By 2010, there were about 101 bike-sharing programs, in about 125 cities around the world, with US having only one [14]. Since 201, however, many US cities started to operate the third generation BSS, such as Boston, New York, Seattle, and Chicago. However, due to the relatively short history of the modern BSS, especially in the US, research on biking mobility and travel patterns remains less documented [11].

### Flow pattern analysis

There have been a number of studies examining BSS functionality and human biking behavior. Corcoran et al. summarized two types of data that are more used in bike sharing research: data capturing stocks (stations) and data capturing flows (trips) [12]. Stock based analysis can reveal the fluctuations in demand and availability in space and time, but hardly reflect the mobility dynamics, such as movement patterns across a city [12]. There were many studies that examine station-use patterns in the previous studies: Froehlich et al., for instance, used 13 weeks of bike station data to investigate user behavior in Barcelona [21]. Etienne and Latifa used model-based clustering method on stations’ departure/arrival counts to explore bike-sharing patterns in Paris [27], associating station neighborhood characteristics with the generated mobility patterns. Faghih-Imani and Eluru developed models to estimate destination preference using distance, land use, built environment, and access to public transportation infrastructure [6]. However, most flow-based analyses were confined to the domain of operational studies, aiming to maximize utility and balance biking networks. However, there have been few studies that examine trip data with the more generic goal of better understanding the dynamics of human spatial mobility [12]. For instance, questions such as the overall biking behavior in a city during peak hours or holidays remain largely unexamined, and to answer these questions, more investigations are required in order to analyze and visualize the complicated citywide trip data.

Flow based analysis, in general, is more analytically complex and difficult to visualize than point based analysis. Lines or curves are usually used to represent flows. However, when flow data becomes bigger, lines representing flows overlap, and the underneath pattern is eclipsed [28]. Several practical approaches have been used to resolve this difficulty, such as sampling or showing a small subset of flow at a time [29, 30]. However, these approaches may either miss some information conveyed by the whole dataset, or lose an overview of patterns revealed by the data [28]. Many recent studies have proposed new methods to analyze and visualize flow data. Zhu and Guo proposed a flow clustering method to extract clusters of similar flows, and reveal summarized flow patterns of taxi trips in Shenzhen, China [13]. Holleczek et al. analyzed and visualized urban mobility patterns using cellphone call data, coupled with public transport smart cards [31]. Taxi trip data and cellphone call data usually contain information about origins and destinations, which resemble the bike-sharing dataset. However, except for bike sharing data visualization competitions in some cities, there have been far fewer papers that analyze massive flow datasets with the more generic goal of understanding overall patterns of bike sharing behaviors [12].

### Over-demand because of asymmetric flows

One feature of bike sharing is the flexibility of checking-out a bike at a station, and returning it to another station. The flexibility of shared bike systems also pose challenges to ensure both bike and dock availability at different places [32]. From previous consumer satisfaction studies, two main problems that promote frustrations are the difficulty to check-out a bike at the start of the journey, due to an empty station, and the difficulty of returning a bike at the destination, due to a full station [33]. These problems can be mitigated by using real time smartphone programs, which inform users of bike and dock availability at a certain location. It is also important to gain a holistic view of station usage in order to improve service. Froehlich et al. used activity score clusters and available bike clusters to identify similar stations, and showed how travel behaviors relate to location, neighborhood, and time of day [21]. Etienne and Latifa used a model-based count series clustering method to detect station service attributes, such as railway stations, parks, employment, and housing, and their temporal demand profile [27]. However, few studies compared these station features across different years. For a young bike-sharing system developed in recent years, it is important to study over-demand patterns in order to better relocate stations and bikes. For instance, from the operator side, if nearby stations have different temporal over-demand patterns, operators can provide incentives to encourage riders to return bikes to nearby stations that have more check-out demand. By doing so, operators can save more bike rebalancing costs. Additional studies are needed to quantify demand and service performance of the system, and thereafter provide guides for system management and potential expansion.

## Data and Method

### Data

We investigated the spatiotemporal patterns of bike sharing behavior in Chicago in 2013 and 2014. Chicago’s BSS are named Divvy, which were launched in late June 2013. The system includes 300 stations and about 3000 bikes. All bikes are available 24 hours, each station has a touchscreen kiosk and docking system which support bikes check-in and check-out using a member key or ride code. Divvy is owned by the Chicago Department of Transportation (CDOT) and operated by Motivate, a company focusing on large-scale bike share systems [34].

Data used in this study was obtained from Divvy website [34]. The dataset includes both trip and station information in both 2013 (from June 27) and 2014. Station data consists of coordinates and dock capacities. Trip data includes massive records about start and end stations, start and end time of trips, trip duration, user types either day pass or annual membership, age and gender information for annual members. To analyze the land use profile associated with stations, we also collected land use inventory from the Chicago Metropolitan Agency for Planning (CMAP). The inventory we used was the 2010 parcel-based land use information. This dataset contains fine-grained land use classes at detailed geographic unit.

### Data preparation

Since the Divvy system was launched in late June 2013, for seasonal consistency, we first subset data in both years from July 1 to December 31. For flow analysis, which will be introduced shortly, we filtered out trips with identical origin and destination (we refer it as same origin-destination rule). Without actual routing information, such trips are not meaningful for flow analysis. Previous study also indicated that such trips might be problematic because of bike malfunctioning and returned back to the original check-out point [6]. In addition, we also deleted trips with duration less than 1 minute; because these trips may not show a common use of the bike-sharing system (we refer it as short trip rule). In 2013, around 5.5 percent and 0.31 percent of trips were deleted because of the same origin-destination rule and the short trip rule respectively. The numbers were 3.7 percent and 0.33 percent in 2014 respectively.

### Bike flow dynamics

To answer the first question “How do the bike flow patterns vary because of time, weekday or weekend, and user groups”, we first identified neighborhood flows for each trip, and then used community detection algorithms to detect trip clusters. Because previous studies pointed out the possible travel pattern difference between weekdays and weekends, and different user types, we first divided our dataset into two groups: trips happened on weekdays or weekends as well as trips made by subscribers or customers.

Specifically, the first step of this analysis was to identify neighboring flows. In this study, we define each trip as a flow from origin to destination. Hence, the neighboring flows of Flow *p* is defined as *NF*_*p*_ = {*F*_*q*_ *∈F*_*a*_|*O*_*q*_ *∈NEI*(*O*_*p*_) & *D*_*q*_ *∈NEI*(*D*_*p*_)}. Where *O* and *D* are the origin and destination of flow *p* respectively. NEI function represents a dichotomous function to select if point *q* is within the neighborhood of point *p*. Zhu and Guo summarized two methods to calculate nearest neighbors for flows, including Euclidian Distance and K-Nearest-Neighbor [13]. In this study, because bike stations in the city periphery were far fewer than city center, we used the Euclidean neighborhoods for clustering analysis to control the search radius for stations in the periphery. Neighborhood flows are defined as:

N(Fp,d)={Fq∈F|Oq∈Ed(Op,Oq) <d&Dq∈Ed(Dp, Dq)<d}

where *O* is the origin and *D* is the destination, *d* is the distance parameter.

It is computation-intensive to calculate each pair of all trips in the dataset. Instead, we first created a station list *S*, which contains the neighbor stations based on Euclidean Neighborhoods for each station. We then created two lists *L*_*O*_ and *L*_*D*_ for each trip *T*_*i*_ to store the trip IDs. These trips should satisfy two criteria:

1. $\;L_{O}\left(F_{i}\right)=\{Fq\in F|Oq\in S_{i}\}$
2. $L_{D}\left(F_{i}\right)=\{Fq\in F|Dq\in S_{i}\}$

where *F* represents all flows in the dataset. *O* and *D* represent origin point and destination point respectively. *S* is the station list created in the previous step.

Then the neighboring flow IDs for *F*_*i*_ is just the intersection of *L*_*O*_ and *L*_*D*_ for flow *F*_*i*_. We calculated the distance between two neighboring flows using Euclidian distance for both origin and destination. Hash mapping for each pair of neighboring flows was used to store the distance.

The second step was to group similar flows into same cluster using graph community detection techniques. We first constructed a graph with neighboring flows being nodes and distance between flows being edge weights. Fastgreedy detection algorithm was used in this study (Formulas 1 and 2). This algorithm aims to optimize modularity functions, which is an index to evaluate the performance of network partitioning [35, 36].

$$Q=\frac{1}{2m}\Sigma_{C\in P}\Sigma_{v,w\in C}(A_{vw}-\frac{k_{v}k_{w}}{2m})$$ Formula 1

$$A_{vw}=\{\begin{array}{ll} 1, & if\;vertices\;v\;and\;w\;are\;connected\\ 0, & \text{Otherwise} \end{array}$$ Formula 2

Where *Q* is the modularity index. Modularity is one index to describe the structure of networks. It was designed to measure the strength of division of a network into smaller communities. Networks with high modularity suggest dense connections between the nodes within communities but sparse connections between nodes in different communities [37]. So the aim of this algorithm is to maximize the value *Q*. *C* is one community in the whole set of *P*. *v*, *w* represent nodes in the network.

Initially, every vertex in the graph was assigned to one community. Then communities were iteratively merged so that each step generated the largest increase of the current modularity. The algorithm stopped when the maximum modularity change became negative. In this study, the outputs of the algorithm were a set of communities, which represented similar flows among all biking trips. We used centroid of origins and destinations for all flows in a community to better visualize clusters.

### Spatiotemporal demands for bikes and docks

To answer the second questions “what are the spatiotemporal over-demands distribution for bikes and docks in 2013 and 2014”, we extracted demand features for each station, compared the demand with the estimated capability for individual station, and clustered similar stations in terms of use patterns. In demand analysis, we subset our dataset into trips happened in weekday and weekend, but not by subscriber and customer because both use types collectively contributed to the station loads.

Specifically, the first step was to define the demand features for bikes and docks. We first defined the analysis time window. Longer time window may lose some variability within each window while shorter time window may introduce too many features that are not representative for the temporal behavior clustering. Prior studies used seven time windows to aggregate biking behavior: 7-9AM, 9AM-1PM, 1-3PM, 3PM-5PM, 5PM-8PM, 8PM-12AM and 12AM-5AM [21]. In this study, we followed this trend and used more homogenous divisions: every two hours being a window except 12am-6am when few riding happens. During each time window, net check-in value was calculated by formula 3.

$$N_{ik}=\Sigma_{t\in W_{i}}IN_{k}-\Sigma_{t\in W_{i}}OUT_{k}$$ Formula 3

Where *i* is one of the ten time-windows, *k* is the station *ID*, *IN* and *OUT* are check-in and check-out numbers respectively. It is important to look at the net value because if large number of check-in and check-out happen simultaneously, the demands for bikes and docks will be cancelled off, which will not introduce additional use pressure for such station. To model the check-in request when all docks are full and check-out request when all docks are empty, we hypothesized the available bike number at the start of each time window as half of the capacity for each station to maximize both check-in and check-out capacity at each station. Hence, the sum of the net check-in value and the hypothesized bike number *S* at station *k*, represents the demand pressure for station *k* at time window *t*. If the *N* is much greater than the capacity, over-demand for docks occurs in window *t*. Similarly, if *N* is a big negative value, the demand for bikes will be significant. This is a simple simulation because operators always redistribute of bikes among different stations. But this index reflects the demand pressures and redistribution cost which will be valuable to understand travel patterns and maintaining systems.

We used agglomerative hierarchical clustering methods to find the stations with similar temporal demands. Agglomerative hierarchical clustering is one type of “bottom up” approaches, which assigns each observation as a cluster first and merges pairs of clusters with similar feature signature into same clusters. The similarity between two clusters can be quantified by different metrics, such as Euclidean distance, Manhattan distance, and Cosine distance. Since each cluster may contain multiple stations, we need to define a function (linkage criteria) of the pairwise distances between two clusters [38]. Results of the clustering process merge stations with similar usage pattern into the same clusters. In this study, the total over-demand numbers for docks and bikes of all weekdays were aggregated to ten time windows (the same window in the previous step). We used cosine distance (Formula 4) as the dissimilarity metric, and complete linkage was used as the linkage criteria.

$$\text{cos}\left(A,\;B\right)=\frac{\Sigma_{i=1}^{n}S_{A}{}_{{}_{i}}\times S_{B}{}_{{}_{i}}}{\sqrt{\Sigma_{i=1}^{n}S_{A}{{}_{{}_{i}}}^{2}}\times \sqrt{\Sigma_{i=1}^{n}S_{B}{{}_{{}_{i}}}^{2}}}$$ Formula 4

Where *cos(A*,*B)* suggests the cosine distance between station *A* and *B*. S_*Ai*_ represents one over-demand variable at time window *i*

To analyze the characteristics of the derived clusters, we investigated the user, directional, and land use profiles for each cluster when over-demand happened. User profile reflected the dominant user characteristics associated with each cluster, including the number of trips, subscribers and customers, gender, age, and ride duration six aspects. Based on the over-demand analysis, we aggregated the user profile for each station at the time when the over-demand happened. Directional profile reflected the dominant ride directions at different time window for stations in each cluster. The direction was calculated based on the angle between the line connecting origin and destination and the horizontal axis. We used rose diagram to represent the overall direction distribution. We also compared the directional profiles for each cluster during the morning and afternoon peak hours and other time windows. Land use profile depicted the major land use types associated with each cluster. In this study, we mainly focused on residential (single-family, single-family attached, and multi-family), commercial (shopping malls, regional & community retail centers, large-site retail, office, and hotel/motel), educational (K-12 education and post-secondary education), vacant (vacant residential land), recreational (open space and primarily recreation) land use, which are the major land use types in the dataset. Consistent with trip clustering analysis, we used the same neighborhood distance to calculate a buffer around each station. The average area of certain kind of land use type within the neighborhood was calculated and aggregated for each cluster. These three profiles were used to infer the functional differences for each cluster.

## Results

### Distance threshold selection

**Fig 2:**
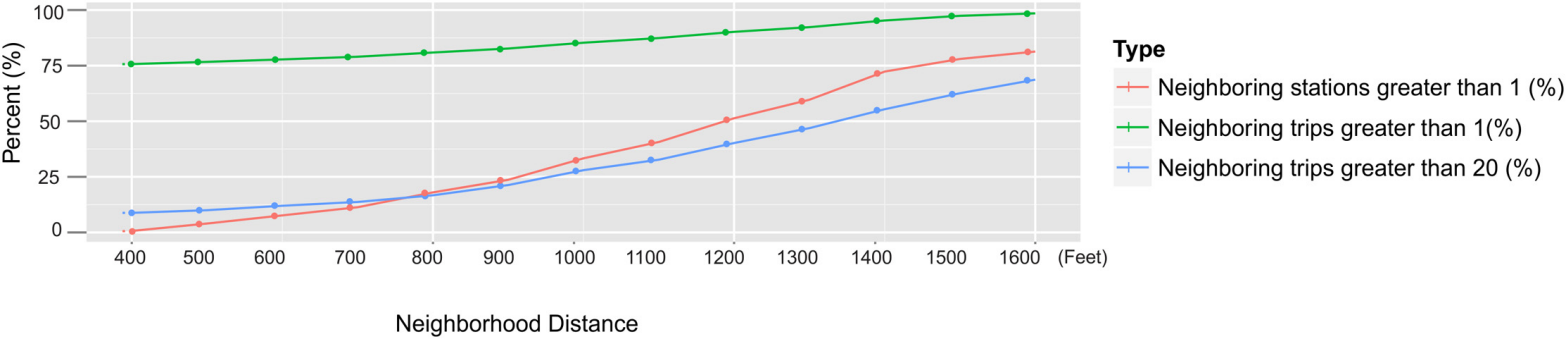
Neighboring stations and trips for the given distance.

The value of distance threshold influences the cluster structure and computation intensity. If *d* is too small, the number of small clusters will be higher. If *d* is too large, it requires more computational power. We compared different distance threshold *d* to find an appropriate value. The dataset is based on subscribers’ weekend trips in 2013. Fig 2 shows the neighboring stations and trips for the given distance. To find the best neighborhood distance threshold, we plotted the distance radius versus three indicators, neighboring stations, trips greater than 1, and trips greater than 20. Based on neighboring stations, we found that the curve leveled off around 426.7 meters (1,400 feet). In addition, we found that when the distance was close to 426.7 meters (1400 feet), almost 75 percent of stations can detect neighbors, and more than 50% of trips had more than 20 neighboring trips. This value should be sufficient for flow clustering analysis, with reasonable computation load. Hence, in the flow dynamics analysis, we select 402 meters (0.25 mile) (1,320 feet, in the range of 1,200–1,400 feet) as the threshold distance.

### Overall trip description

**Fig 3:**
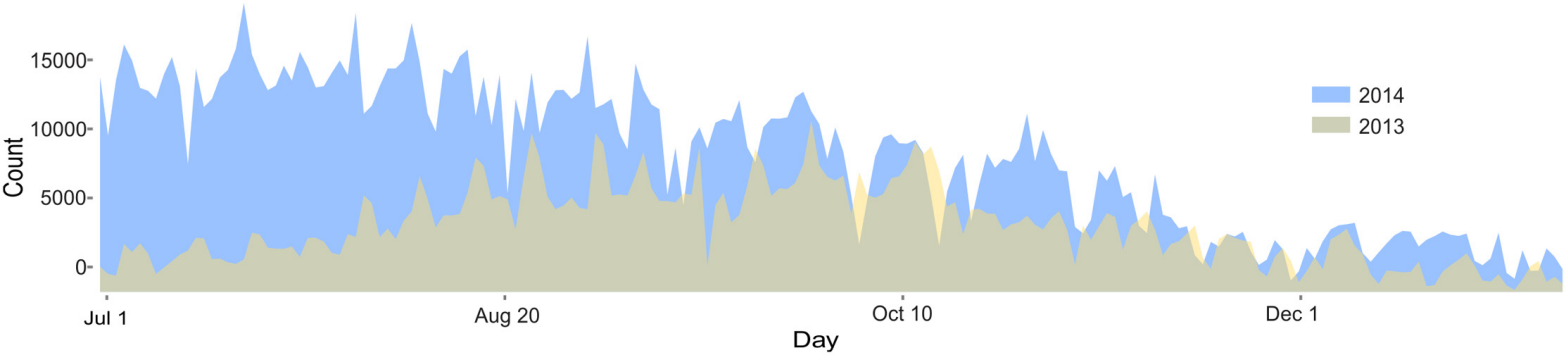
Comparison of Divvy daily usage between two years.

From July 1 to December 31, there were 756,670 trips in 2013 and 1,548,935 in 2014. Among these trips, in 2013, 53.3 percent of trips were made by subscribers, who had unlimited 30-minute bike trips throughout the year. In 2014, this number increased to 69.2 percent, suggesting the BSS gained more regular users. Fig 3 shows the overall pattern of Divvy system usage over the two years. The total rides by customers and subscribers increased from 2013 to 2014, especially significant in the time window July to August, when Divvy was launched in 2013. Seasonal differences were also obvious. There were significantly more rides in summer than in winter (Fig 3).

**Fig 4:**
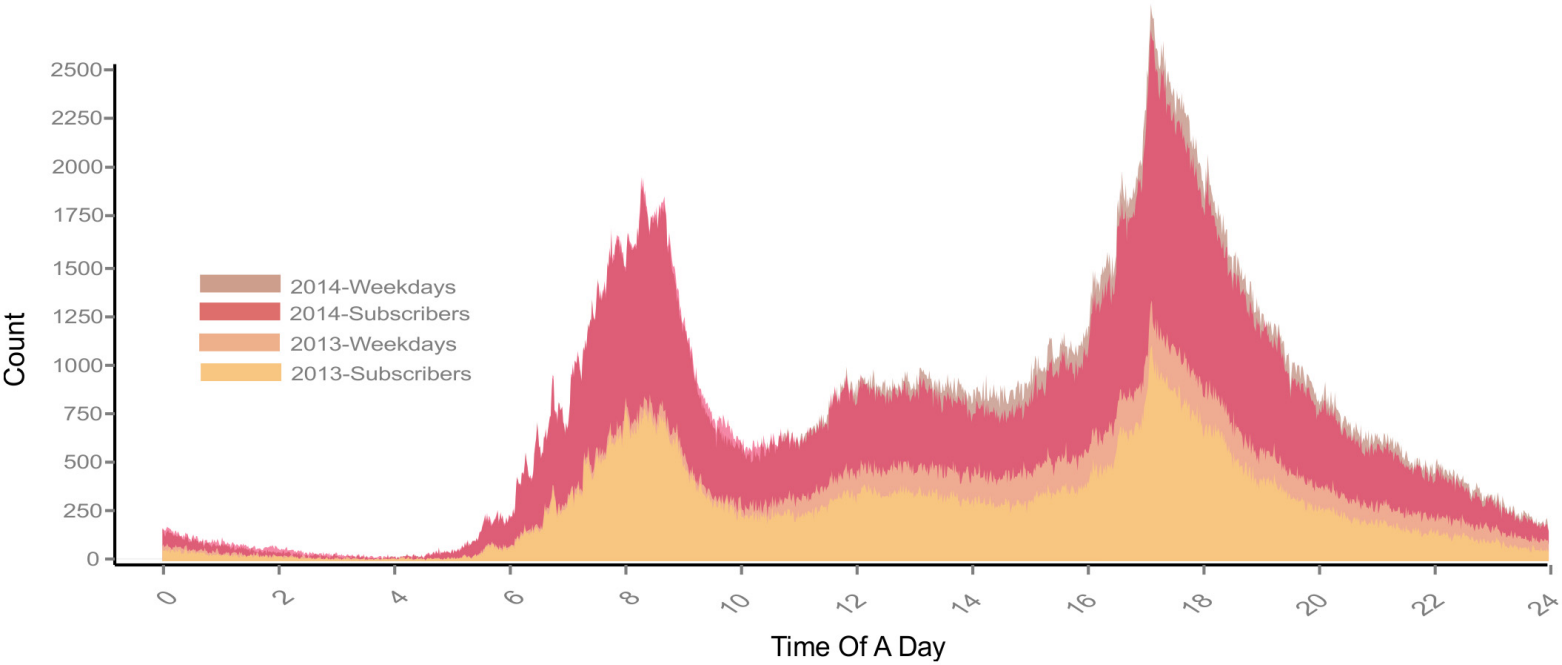
Subscribers’ biking behavior at different time periods.

**Fig 5:**
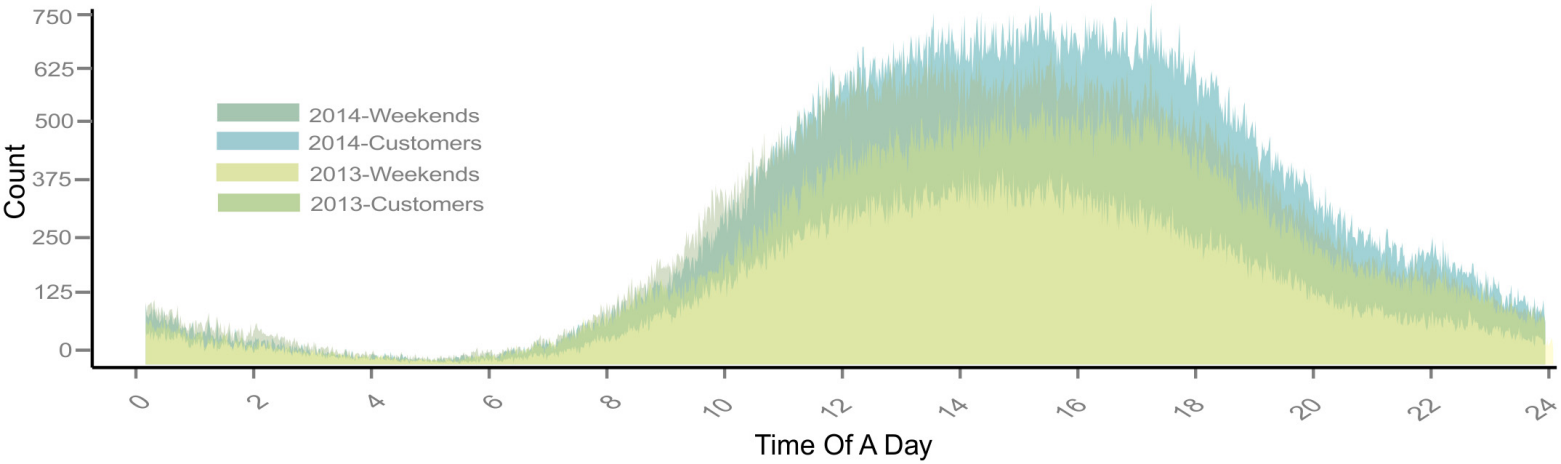
Customer’ biking behavior at different time periods.

We also compared the customers’ and subscribers’ travel patterns at different times of a day. Overall, subscribers’ travel patterns were similar to overall weekday travel patterns. Usage peaked at rush hours, around 8am and 6pm. Moreover, the spike during the afternoon rush hour was sharper than in morning, which suggested that traffic concentration in the afternoon was more significant (Fig 4). Weekend BSS usage was much less than on weekdays. Comparing user type, customers were much more frequent than subscribers. From this, we can infer that subscriber uses were more commute-oriented, and customer uses were more recreation-oriented. Most customer and weekend use concentrated and evenly distributed between 10am and 8pm (Fig 5).

**Fig 6:**
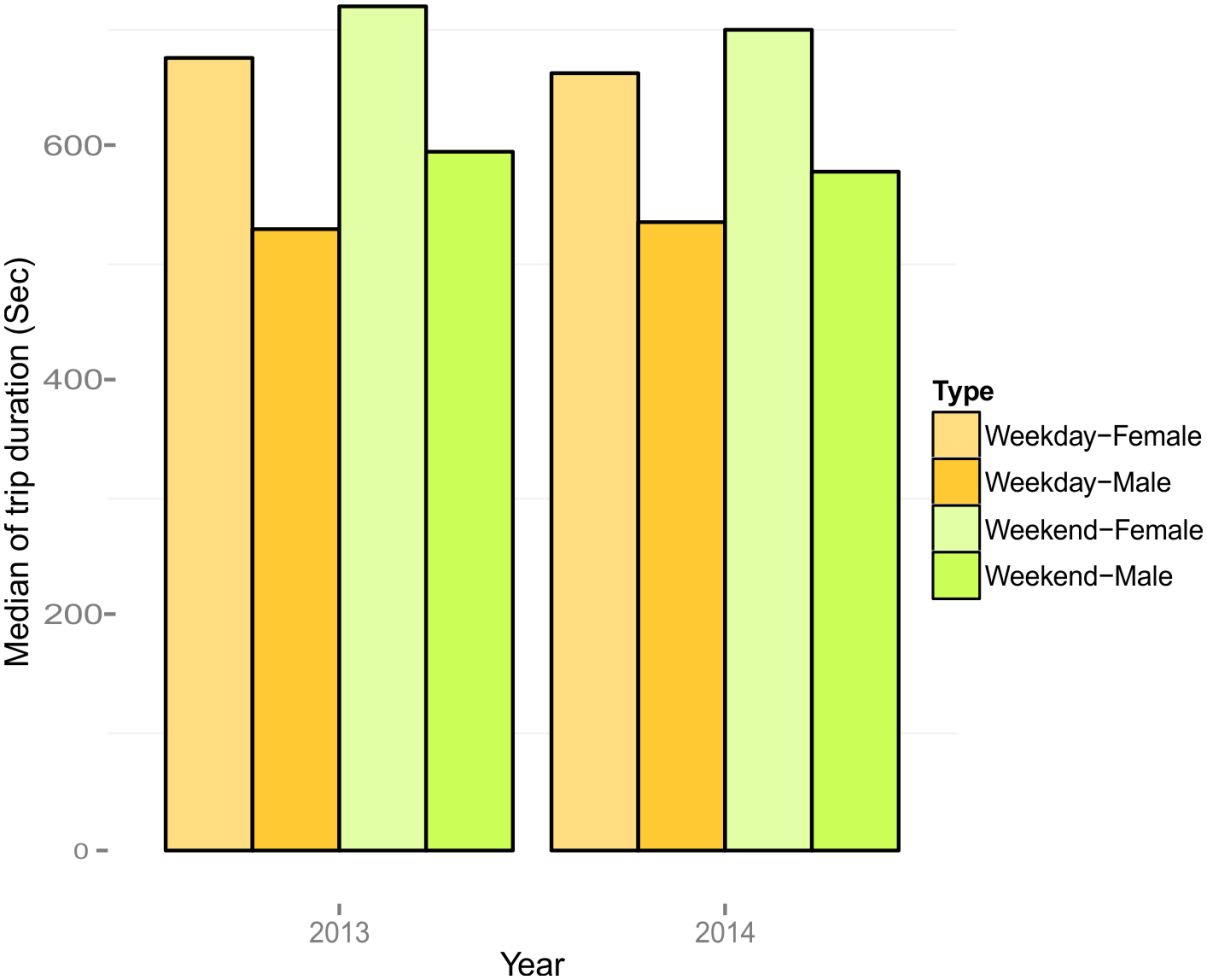
Median of trip duration for males and females in 2013 and 2014.

People rode longer during weekdays than weekends, but surprisingly, females in general rode longer in duration than males (Fig 6). We also compared the distance traveled between females and males. In 2013, females on average rode 113.4 meters (372 feet) and 210.3 meters (690 feet) more than males did on weekends and weekdays, respectively.

### Bike flow dynamics

**Fig 7:**
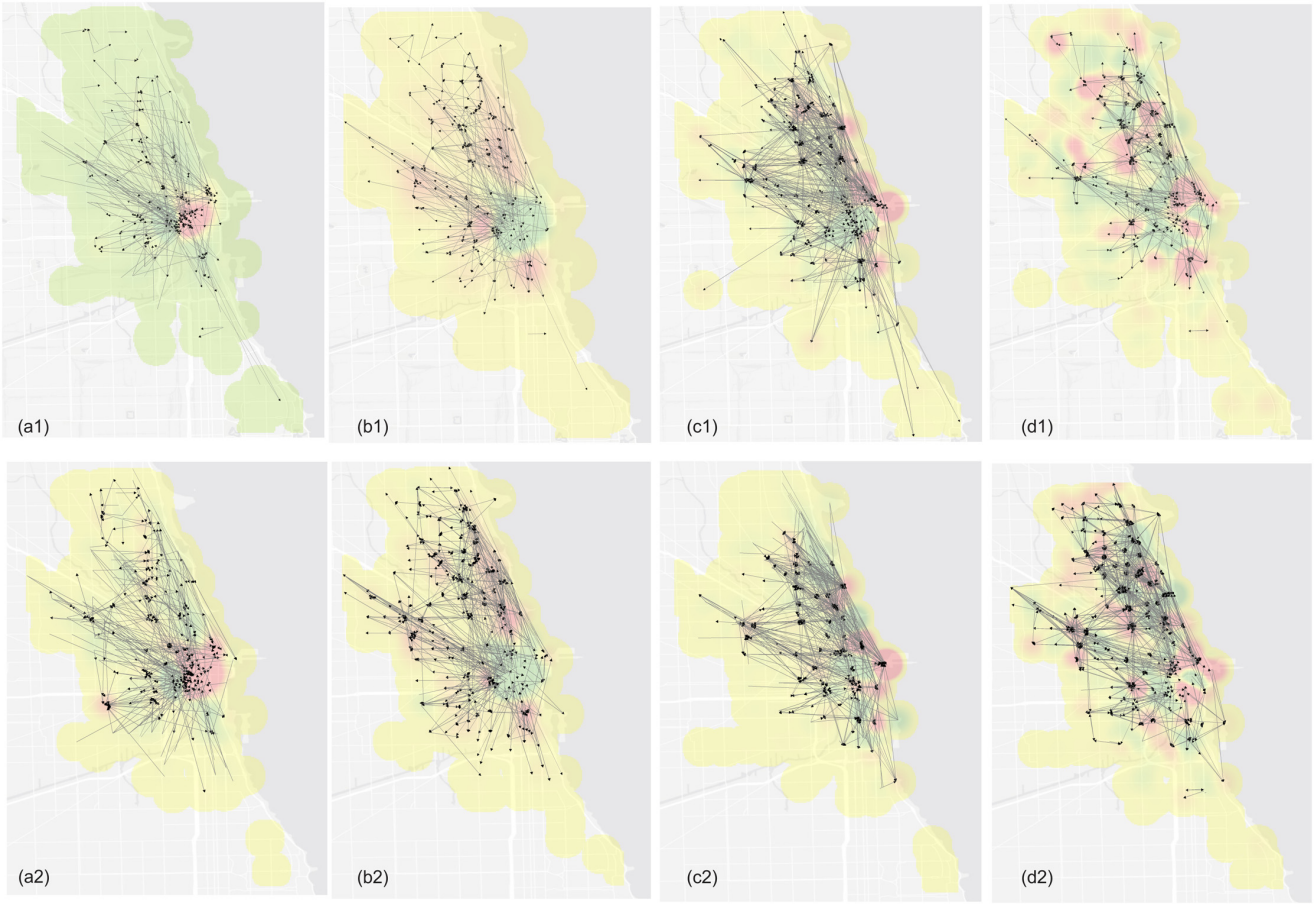
(a) Flow clusters for morning peak hours on weekdays. (b) Flow clusters for afternoon peak hours on weekdays. (c) Customers’ travel patterns during weekend. (d) Subscribers’ travel patterns during weekend. Background color represents the flow-in density. Red color means trips converge while green color means trips flow away. Group 1 represents 2013 and Group 2 represents 2014.

The trip dataset contained large amounts of information. Plotting only ten percent or one percent of trips still generated a cluttered map (Fig 1). In contrast, results of our bike flow clustering demonstrated meaningful patterns. We compared bike flow patterns in the morning and afternoon peak hours on weekdays in 2013 and 2014. Weekends did not show any travel peak hours, but customers and subscribers exhibited different biking behaviors. Fig 7 showed results of the comparisons. Fig 7a1 demonstrated people’s travel patterns during weekday morning peak hours. We detected 260 clusters with size greater than 20. Intuitively, the inbound trips were dominant. A majority of trips travelled into the downtown areas. Many long travel clusters were also discernable, such as flow clusters from South Chicago to downtown areas. The average Euclidean travel distance among all stations was 1.96 miles. Fig 7b1 showed biking patterns of afternoon peak hours during weekdays. As opposed to morning patterns, outbound trends were obvious. The green color suggested many bikes were checked-out in the downtown area, and traveled to surrounding areas, which were represented by the red color. Interestingly, during weekends, customers and subscribers’ biking behaviors exhibited different patterns. Fig 7c1 showed customers’ travel patterns. We can clearly identify many trip clustered into the Downtown, Navy Pier, and a belt along Lake Michigan, where recreational bike lanes were concentrated. The average flow cluster distance for customers was 2.2 miles, which was significantly higher than for subscribers. Unlike customers, subscribers’ weekend travels did not show particular patterns (Fig 7d1). Although some clusters still flow to downtown, the travel patterns were generally more diverse than that of customers.

The overall flow patterns were similar in 2013 and 2014. Morning and afternoon peak hours revealed similar strong inbound and outbound clusters. Flow hotspots for subscribers and customers had similar spatial patterns. The main difference was the cluster intensity. Comparing cluster numbers with the size greater than 20, there were 260 flow clusters in 2013. This number increased to 378 in 2014. This reflected the fact that although a significant amount of people started using the Divvy system, overall usage patterns remained the same in two years.

### Spatiotemporal demands for bikes and docks

**Fig 8:**
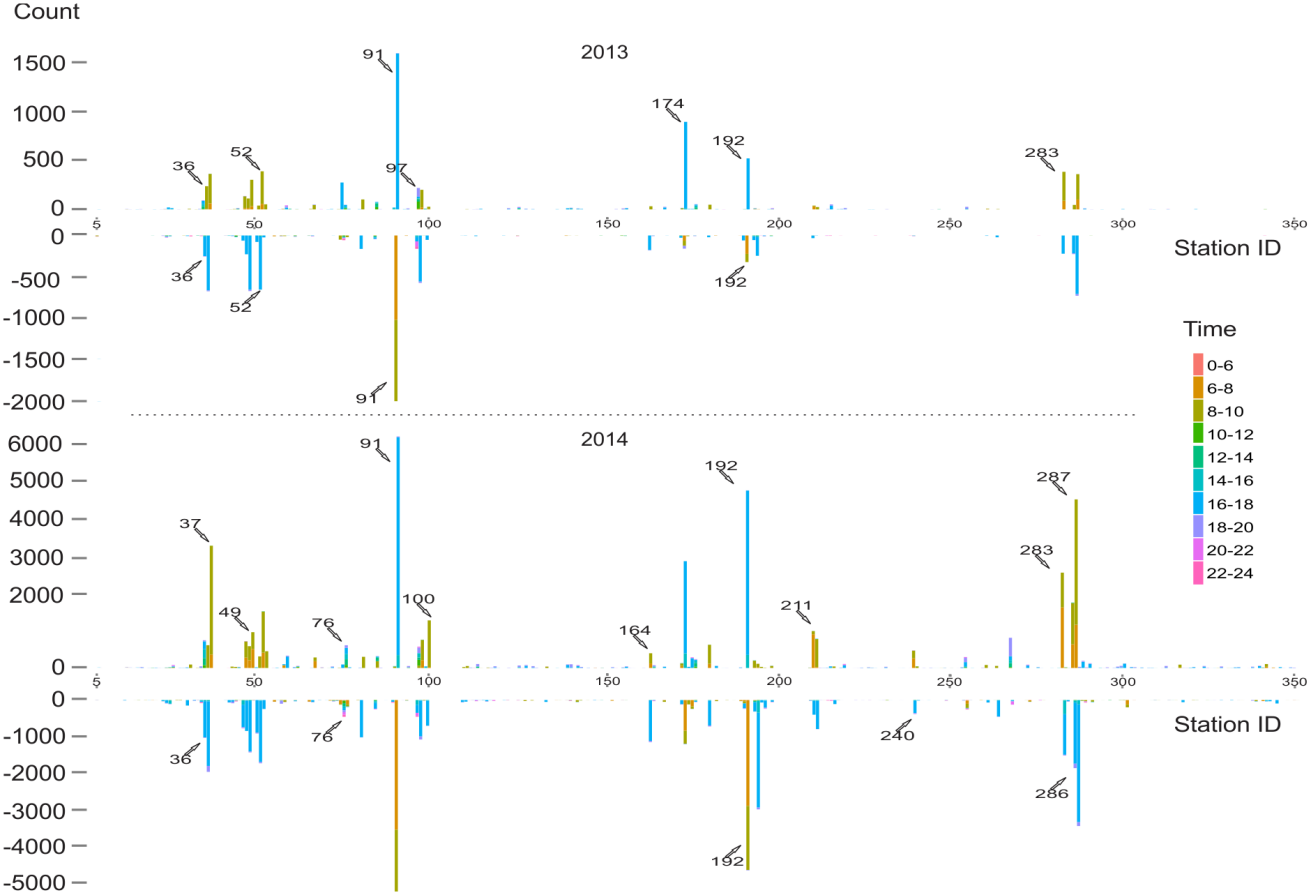
Demands for dock and bike that exceed the hypothesized service capacity. Counts above zero represent the total number of bikes that are not able to check in due to full docks. Counts below zero represent the total number of failed attempts to check out bikes due to empty docks. Colors represent different time windows.

To investigate spatiotemporal over-demands for bikes and docks in 2013 and 2014, we modeled the total cases of dock overload and bike insufficiency for each station in both years. Fig 8 shows the results. Demand for bike check-in and check-out in 2014 was much higher than in 2013, especially for several stations, such as #100, #164, and #212, where almost no over-demand situations happened in 2013. Second, we found that several stations experienced high dock and bike demands at different time windows, such as at stations #91 and #192. These stations were close to transit stations, and over-demand patterns closely correlated with work commute. Third, interestingly, we noticed that several stations experience greater check-in pressure from 10am-14pm during weekdays, such as #76, #97, #35, #177, and #268. Almost all stations were close to the shores of Lake Michigan.

**Fig 9:**
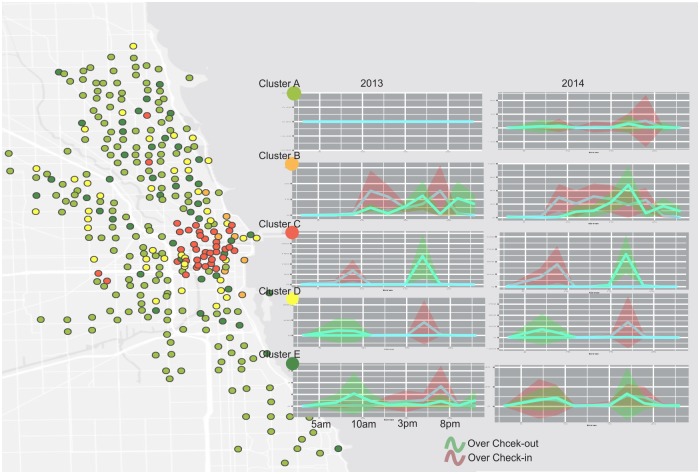
Spatiotemporal clusters for bike and dock over-demand. Stations were classified into five classes. Each cluster has its own temporal use pattern. Red curve represents the over check-in pattern and green curve represents the over check-out pattern.

To find the spatiotemporal patterns of over-demand for bikes and docks, we also conducted hierarchical clustering analysis. Based on the spatiotemporal demands analysis, we found that stations exhibit several usage patterns: strong morning peak check-in, strong morning peak check-out, strong non-peak hour use, mixed use, and low use. Hence we used five classes to clustering the over-demand patterns. To better illustrate the temporal use patterns for each type of station, we plotted a temporal use spectrum based on the over-demand rate for docks and bikes (Fig 9). The red curve represented a strong demand for check-in requests, and the green curve represented a strong check-out request. The map showed the spatial distribution of these five clusters. Cluster A represented a relatively low use cluster. In 2013, stations in Cluster A did not experience any overload problems, while this happened to some stations during peak hours in 2014. Cluster B exhibited a strong non-peak hour use. Locations of these stations were close to the Chicago Museum Campus, Millennium Park, and Magnificent Mile. We thus suspect this cluster might be related to recreational travels. Cluster C and Cluster D showed opposite temporal usage trends. Cluster C showed a strong check-in trend in the morning peak hours, and a strong check-out trend in the afternoon peak hours. We estimated these stations might be associated with workplace. On the other hand, many bikes checked-out in the morning, and returned in the afternoon. These stations were more associated with residence. The temporal use signatures of Cluster E were different from the rest of the classes. The use rate was higher than Cluster A, but the demand for check-in and check-out was mixed. Demand for bike rebalancing for this type of station was not as strong as other types.

To further investigate the characteristics associated with each cluster, we conducted user, directional, and land use profile analysis. Table 1 reflected the user profile of each cluster in 2013 and 2014. Cluster C and D were associated with the most trips and subscribers in both 2013 and 2014, suggesting these stations were used more for commuting purposes. Cluster B was the only class of which the number of customers was much higher than that of subscriber’s. In addition, the average trip duration for Cluster B was also significantly longer than the rest of Clusters. The user profile suggested this cluster was more associated with recreational trips. Based on the over-demand pattern, Cluster A did not show any over-demand cases in 2013 while the cases emerged in 2014. The user profiles of Cluster A in 2014 and Cluster E were similar to C & D but with much fewer trips. The user gender did not show obvious patterns among clusters and between two years. Comparing two years, in general, increasing number of younger people started to use the Divvy Systems in 2014 across five clusters.

**Table 1:** User profile of each cluster when the modeled over-demand happened in 2013 and 2014.

Year	Cluster	Customer #	Subscriber # (%)	Trip #	Ave. Duration (Sec)	Male #	Female # (%)	Median Age
2013	A	0	0 (N/A)	0	N/A	0	0 (N/A)	N/A
	B	38283	12602 (24.8)	50885	1607	9832	2767 (22.0)	34
	C	21826	95771 (81.4)	117597	864	78992	16777 (17.5)	36
	D	36453	58682 (61.7)	95135	1078	49481	9199 (15.7)	38
	E	12952	21937 (62.9)	34889	1109	17165	4772 (21.8)	33
2014	A	37186	146586 (79.8)	183772	909	107532	39058 (26.6)	31
	B	59182	38498 (39.4)	97680	1363	27949	10547 (27.4)	34
	C	32698	271523 (89.3)	304221	772	219220	52251 (19.2)	34
	D	76517	214345 (73.7)	290862	939	170362	43970 (20.5)	35
	E	38252	109640 (74.1)	147892	937	82627	27020 (24.6)	32

**Fig 10:**
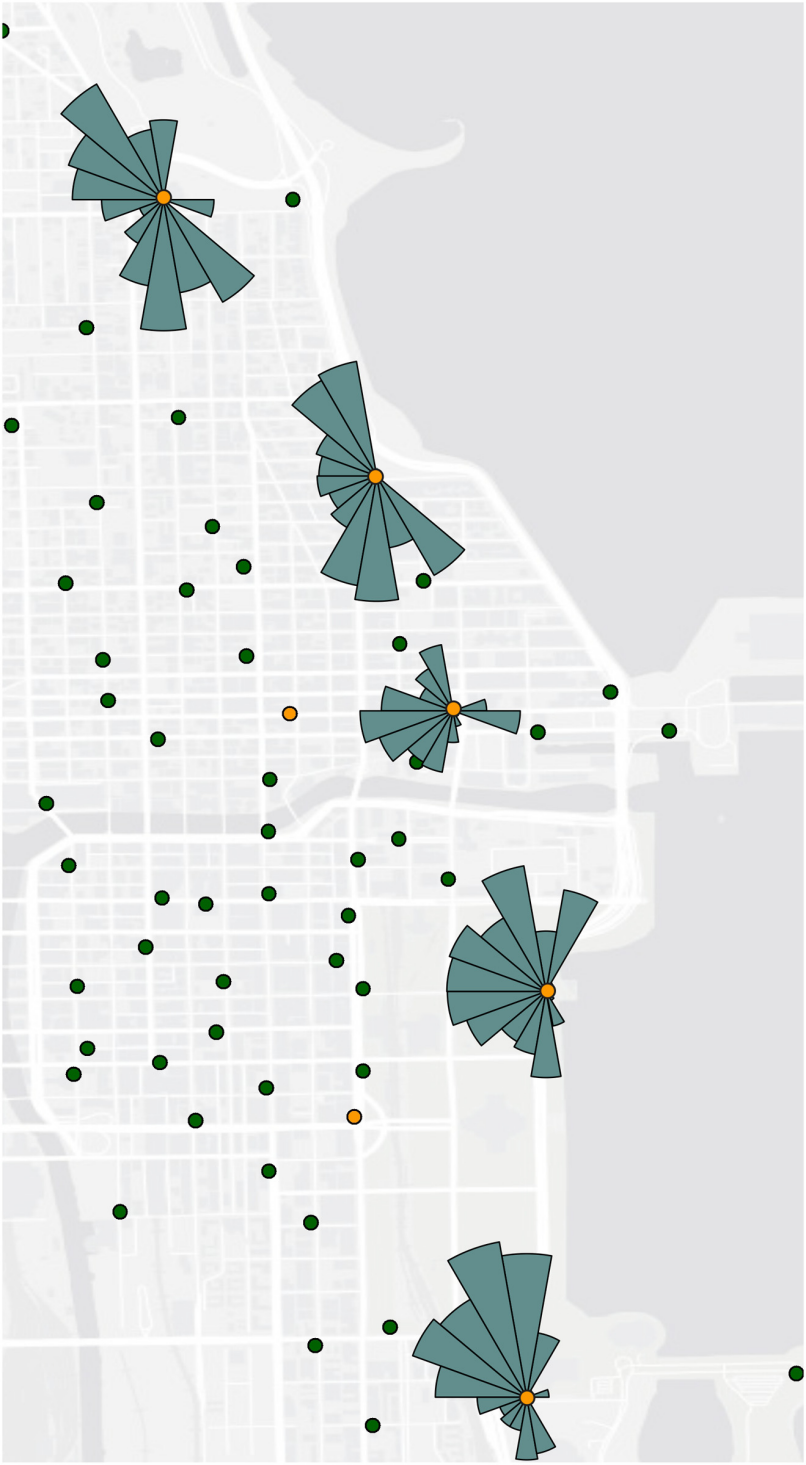
The over-demand directional profile for stations in Cluster B in the evening.

**Fig 11:**
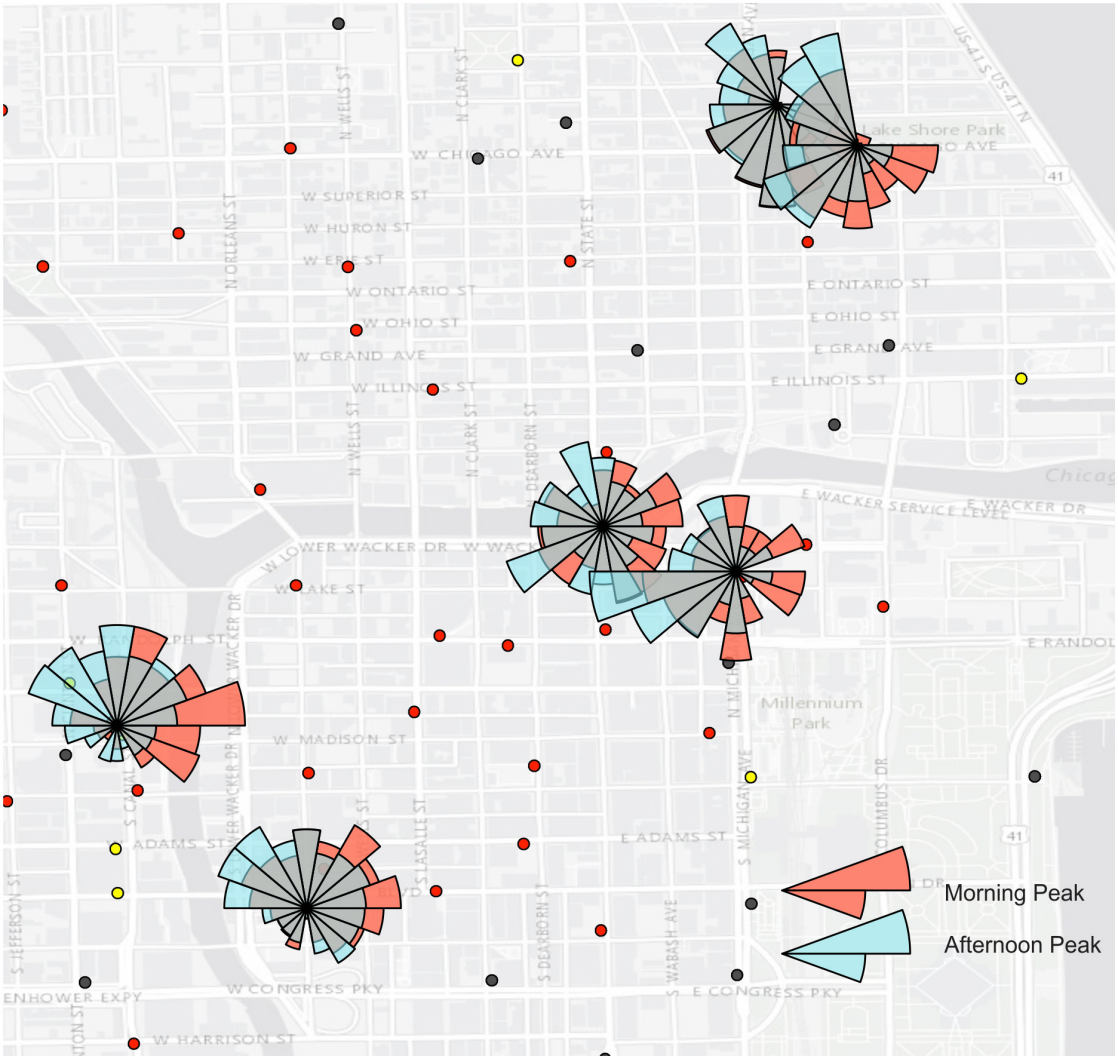
The stations had significantly more check-out records than check-in records during both morning and afternoon peaks in Cluster C and D.

Directional profile revealed the dominant ride directions at different time window for stations in each cluster. As with the flow cluster analysis, we can find a strong inbound direction for Cluster D during the morning peaks and a strong outbound trend for Cluster C during the afternoon peaks. In addition, we also noticed some interesting patterns. After 8pm, most clusters become less busy. However, most stations in Cluster B were still active and revealed some directional patterns. Fig 10 reflected the over-demand directional profile for stations in Cluster B after 8pm. The trips exhibited a strong north-south flow direction along the coast. These trips resembled recreational rides along the coastal park close to Michigan Lake, which was a hotspot for activities in the evening. In addition, in Cluster C and D, there were some stations had greater check-out records than check-in records during both morning and afternoon peaks (Fig 11). In the morning, bikes in these stations were checked out and ridden to most directions on the east side of the Chicago River. The flow directions implied the activity hotspots during the work time. During the afternoon peak hours, however, bikes of these stations were mostly ridden to the west and north directions. Fewer bikers moved the south direction. Cluster A and E did not show particular directional profiles.

We also calculated the land use profile for each cluster. Cluster C was mostly associated with commercial land use. The average area of commercial land use surrounding stations in Cluster C was 152576.8 square meters. Comparing to Cluster B and C, more stations in Clusters A, D, E were closer to residential area. Although stations in Cluster A contained relatively fewer trips, their neighborhood covered a lot of educational land use. Most stations in Cluster C were located in downtown areas. For those stations in Cluster C but not in downtown areas, they were mostly related to educational facilities, for instance, University of Illinois at Chicago in the west and DePaul University in the north. For recreational land use, Cluster B accounted for 48.8%. This also confirmed that many trips connecting stations in Cluster B were recreational-related. A good deal of vacant residential land was in the neighborhood of Cluster A. This may partly explain the relatively lower usage for Cluster A. The land use profile further demonstrated the characteristics of each cluster (Table 2).

**Table 2:** The average area of certain land use type in station buffers. Unit of the area is square meter. Numbers in the parenthesis represent the percentage of land use of certain kind among five clusters.

Cluster	Commercial	Residential	Educational	Recreational	Vacant Residential
A	12685.2 (4.7)	194040.1 (27.5)	45156.6 (38.0)	61738.0 (7.7)	8341.3 (46.4)
B	48418.8 (17.8)	91234.4 (12.9)	8565.9 (7.2)	391928.4 (48.8)	52.5 (0.3)
C	152576.8 (56.0)	62178.2 (8.8)	18682.1 (15.7)	74155.7 (9.2)	3339.3 (18.6)
D	36189.8 (13.3)	174675.6 (24.7)	27093.8 (22.8)	117305.4 (14.6)	1775.7 (9.9)
E	22509.11 (8.3)	184401.8 (26.1)	19477.8 (16.4)	157228.1 (19.6)	4454.7 (24.8)

## Discussion

The bike sharing system in the US developed rapidly in recent years. From usage comparisons in Chicago, the overall trips made from July to December have doubled from 2013 to 2014. Among all trips, the proportion from subscribers increased from 53.3% to 69.2% from 2013 to 2014. Consistent with prior study, we found that women rode longer than men. By comparing the average origin and destination distances between women and men, we found that females on average rode 113.4 meters (372 feet) and 210.3 meters (690 feet) more than males did on weekends and weekdays, respectively. In Chicago, only about 21% of members are women [39]. It is possible that most women who use the BSS are regular users, and are more interested in physical activity. These women are thus more likely to pursue longer trips [39]. It would be interesting to compare gender differences in other cities.

This study investigated bike-sharing patterns from both flow and station perspectives. From flow pattern analysis, we developed an effective approach to extract meaningful clusters from massive amounts of travel data. The derived travel patterns were valuable to provide reference and evidence for sustainable transportation planning. For instance, we found that there were long distance biking clusters connecting south Chicago and downtown areas. One reason for this long distance ride was the well-connected and standalone bike lanes along the Lake Michigan. People are willing to bike longer for both transportation and recreation purposes if the urban environment is safe and encouraging. From a station demand analysis, this paper also examined temporal patterns of over-demand for bikes and docks. Results of station demand analysis were valuable in order to better operate the sharing systems. For instance, by comparing system use between two years, we observed that the modeled over-demand rate at several stations grew significantly faster from 2013 to 2014. Operators may need to consider additional redistribution approaches in order to handle the disproportional increase. In addition, the presented methods were able to efficiently handle large amounts of flow data, and extract underlying patterns of travel behaviors. Results clearly revealed distinct travel amounts, direction, and concentrations at different times of day.

The asymmetry feature of trips in a network (disproportionate amount of trips between two stations at certain time) may cause insufficient services for docks or bikes. This study used a simple but effective model to estimate over-demand across space and time. Results from station clustering suggested that even geographically close stations might exhibit different demand patterns. The system operator may propose incentives to encourage people to return bikes to close stations, where demand is higher during certain time windows. For instance, station A at Canal St. & Madison St. and station B at Canal St. & Monroe St. Although these two stations were only several blocks away, their over-demand patterns were very different. Operators can provide incentives to attract users to return bikes to station B, rather than station A, during morning peak hours to reduce rebalancing costs.

Insight gained from this study can be extended to other cities. In this study, we found dominant inbound flows in the morning and outbound flows in the afternoon peak hours. This corresponded to the zonal urban structure (single-center) of Chicago. This general flow pattern may apply to cities with similar concentric zone urban structure. However, cities with other urban structures such as Sector Model or Multiple Nuclei Model can apply the flow clustering method to investigate the relationship between biking patterns and city structures. Second, in this study, we observed the land use profile of station clusters were related to the BSS use patterns. For instance, stations close to universities may boost students’ physical activity while stations near parks and recreational areas may involve more customer usage. The different user groups may generate unique mobility patterns. These factors should be considered when deploying the systems. Also, future studies can investigate if the mixed land use is associated with more biking activities. Third, this study also examined the spatial-temporal usage difference for subscriber and customers. The knowledge gained from analyzing the biking mobility could be very helpful to identify complementary patterns, which will be useful to optimize the systems. For instance, currently many BSS employ a flat rate. Planners might consider developing floating rate systems to encourage people to return bikes to the desirable locations or encourage customers use less when and where utilitarian demand is high. Methods used in this study can be also applied in different city context. The over-demand model can be applied in other cities to anticipate usage patterns. This would be helpful for better rebalancing bikes and more effective budgeting. In addition, the clustering methods can be applied to other broader problems involving data with origin-destination forms. For instance, such method can be used to study the taxi mobility patterns if the dataset contains pick-up and drop-off locations.

We plan to extend current study by integrating public transit data, and data for other types of transport. First, as city transportation data becomes more available, it offers great opportunities to crowd-source multiple datasets, such as public transit data or car sharing data, with which to examine city transportation patterns. Bike sharing data can be integrated with other public transit data to harmonize bike-sharing infrastructure with public transit transportation service. In our study, we found that the stations with the most over-demand during weekdays were actually not located at the center of the business district, but rather very close to Union Station and subway stations. Many commuters use public transit to traverse longer distances, and then connect with Divvy Bikes to get to their final destinations. It would be interesting to jointly look at travel patterns of public transit and bike sharing, in order to create a more seamless multi-modal transportation system. City planners can expand infrastructure to smooth connections between subway and bike stations. Policy-makers can consider an integrated or discounted fair to promote joint use of these two systems.

## Conclusion

This study investigated the spatiotemporal biking pattern in Chicago by analyzing BSS data from July to December in 2013 and 2014. Our work mainly contributes to the growing literature in bike-sharing systems in two aspects. First, we detected and visualized flow clusters by examining massive individual trips. Results clearly reflected major spatiotemporal bike flow patterns in Chicago. Second, this study also analyzed station over-demand patterns across the city. Results of hierarchical clustering revealed the spatial distribution of stations with different temporal use signatures. We also used user, directional, and land use profiles to investigate the functional characteristics of the derived clusters. The proposed methods can be applied to other cities in order to study citywide biking behavior, and mobility patterns. The results are useful in planning future stations, and developing incentives to better rebalance bike service. We plan to extend our current study by integrating public transit data to create a more seamless multi-modal transportation system.

## References

[1] Goodman A, Cheshire J. Inequalities in the London bicycle sharing system revisited: impacts of extending the scheme to poorer areas but then doubling prices. Journal of Transport Geography. 2014;41:272–9.

[2] Maizlish N, Woodcock J, Co S, Ostro B, Fanai A, Fairley D. Health cobenefits and transportation-related reductions in greenhouse gas emissions in the San Francisco Bay area. American Journal of Public Health. 2013;103(4):703–9. doi: 10.2105/AJPH.2012.300939 23409903 10.2105/AJPH.2012.300939 PMC3673232

[3] Guell C, Panter J, Jones N, Ogilvie D. Towards a differentiated understanding of active travel behaviour: using social theory to explore everyday commuting. Social Science & Medicine. 2012;75(1):233–9. 22486840 10.1016/j.socscimed.2012.01.038 PMC3611601

[4] Shaheen S, Martin E, Cohen A. Public bikesharing and modal shift behavior: a comparative study of early bikesharing systems in North America. Int J Transport. 2013;1(1):35–53.

[5] Darren F. National Household Travel Survey—short trips analysis 2010 [21 May 2015]. Available: http://www.bikeleague.org/content/national-household-travel-survey-short-trips-analysis.

[6] Faghih-Imani A, Eluru N. Analyzing Destination Choice Preferences in Bicycle Sharing Systems: An Investigation of Chicago’s Divvy System. 2014.

[7] Fishman E, Washington S, Haworth N. Bike share: a synthesis of the literature. Transport reviews. 2013;33(2):148–65.

[8] O’Brien O, Cheshire J, Batty M. Mining bicycle sharing data for generating insights into sustainable transport systems. Journal of Transport Geography. 2014;34:262–73.

[9] Beecham R, Wood J. Exploring gendered cycling behaviours within a large-scale behavioural data-set. Transportation Planning and Technology. 2014;37(1):83–97.

[10] Vogel M, Hamon R, Lozenguez G, Merchez L, Abry P, Barnier J, et al From bicycle sharing system movements to users: a typology of Vélo’v cyclists in Lyon based on large-scale behavioural dataset. Journal of Transport Geography. 2014;41:280–91.

[11] Jäppinen S, Toivonen T, Salonen M. Modelling the potential effect of shared bicycles on public transport travel times in Greater Helsinki: An open data approach. Applied Geography. 2013;43:13–24.

[12] Corcoran J, Li T, Rohde D, Charles-Edwards E, Mateo-Babiano D. Spatio-temporal patterns of a Public Bicycle Sharing Program: the effect of weather and calendar events. Journal of Transport Geography. 2014;41:292–305.

[13] Zhu X, Guo D. Mapping large spatial flow data with hierarchical clustering. Transactions in GIS. 2014;18(3):421–35.

[14] Shaheen SA, Guzman S, Zhang H. Bikesharing in Europe, the Americas, and Asia. Transportation Research Record: Journal of the Transportation Research Board. 2010;2143(1):159–67.

[15] ITDP. The Bike-Share Planning Guide 2013. Available from: https://www.itdp.org/the-bike-share-planning-guide-2/.

[16] García-Palomares JC, Gutiérrez J, Latorre M. Optimizing the location of stations in bike-sharing programs: a GIS approach. Applied Geography. 2012;35(1):235–46.

[17] Bonnette B. The Implementation of a Public-Use Bicycle Program in Philadelphia. 2007.

[18] Nadal L. Bike sharing sweeps Paris off its feet. Sustainable transport. 2007(19).

[19] Jensen P, Rouquier J-B, Ovtracht N, Robardet C. Characterizing the speed and paths of shared bicycle use in Lyon. Transportation research part D: transport and environment. 2010;15(8):522–4.

[20] Côme E, Randriamanamihaga A, Oukhellou L, editors. Spatio-temporal usage pattern analysis of the Paris Shared Bicycle Scheme: a data mining approach. Transport Research Arena (TRA) 5th Conference: Transport Solutions from Research to Deployment; 2014.

[21] Froehlich J, Neumann J, Oliver N, editors. Sensing and Predicting the Pulse of the City through Shared Bicycling. IJCAI; 2009.

[22] Midgley P. The role of smart bike-sharing systems in urban mobility. Journeys. 2009;2:23–31.

[23] Shaheen S, Guzman S, Zhang H. Bikesharing in Europe, the Americas, and Asia: past, present, and future. Transportation Research Record: Journal of the Transportation Research Board. 2010(2143):159–67.

[24] Zhang L, Zhang J, Duan Z-y, Bryde D. Sustainable bike-sharing systems: characteristics and commonalities across cases in urban China. Journal of Cleaner Production. 2015;97:124–33.

[25] Shaheen S, Zhang H, Martin E, Guzman S. China's Hangzhou public bicycle: understanding early adoption and behavioral response to bikesharing. Transportation Research Record: Journal of the Transportation Research Board. 2011(2247):33–41.

[26] Zhao J, Wang J, Deng W. Exploring bikesharing travel time and trip chain by gender and day of the week. Transportation Research Part C: Emerging Technologies. 2015.

[27] Etienne C, Latifa O. Model-Based Count Series Clustering for Bike Sharing System Usage Mining: A Case Study with the Vélib’System of Paris. ACM Transactions on Intelligent Systems and Technology (TIST). 2014;5(3):39.

[28] Guo D. Flow mapping and multivariate visualization of large spatial interaction data. Visualization and Computer Graphics, IEEE Transactions on. 2009;15(6):1041–8. 10.1109/TVCG.2009.143 19834170

[29] Rae A. From spatial interaction data to spatial interaction information? Geovisualisation and spatial structures of migration from the 2001 UK census. Computers, Environment and Urban Systems. 2009;33(3):161–78.

[30] Woodruff A. Hubway snapshot 2012. Available: http://bostonography.com/hubwaymap.

[31] Holleczek T, Yu L, Lee JK, Senn O, Kloeckl K, Ratti C, et al. Digital breadcrumbs: Detecting urban mobility patterns and transport mode choices from cellphone networks. arXiv preprint arXiv:13086705. 2013.

[32] Nair R, Miller-Hooks E, Hampshire RC, Bušić A. Large-scale vehicle sharing systems: analysis of Vélib'. International Journal of Sustainable Transportation. 2013;7(1):85–106.

[33] Kaltenbrunner A, Meza R, Grivolla J, Codina J, Banchs R. Urban cycles and mobility patterns: Exploring and predicting trends in a bicycle-based public transport system. Pervasive and Mobile Computing. 2010;6(4):455–66.

[34] Divvy. 2013 [8 May 2015]. Available: https://www.divvybikes.com.

[35] Newman ME, Girvan M. Finding and evaluating community structure in networks. Physical review E. 2004;69(2):026113. 10.1103/PhysRevE.69.026113 14995526

[36] Clauset A, Newman ME, Moore C. Finding community structure in very large networks. Physical review E. 2004;70(6):066111. 10.1103/PhysRevE.70.066111 15697438

[37] Fortunato S. Community detection in graphs. Physics Reports. 2010;486(3):75–174.

[38] Han J, Kamber M, Pei J. Data mining, southeast asia edition: Concepts and techniques: Morgan kaufmann; 2006.

[39] Faghih-Imani A, Eluru N. Analysing bicycle-sharing system user destination choice preferences: Chicago’s Divvy system. Journal of Transport Geography. 2015;44:53–64.

